# Healthcare costs of congenital cytomegalovirus (cCMV) disease in infants during the first two years of life: a retrospective German claims database analysis

**DOI:** 10.1186/s12962-022-00411-x

**Published:** 2023-01-23

**Authors:** Anna-Janina Stephan, Marion de Lepper, Regine Wölle, Agnes Luzak, Wei Wang, Christian Jacob, Kim Maren Schneider, Horst Buxmann, Rangmar Goelz, Klaus Hamprecht, Peter Kummer, Susanne Modrow, Wolfgang Greiner, Miriam Reuschenbach

**Affiliations:** 1 Department of Market Access, MSD Sharp & Dohme GmbH, Munich, Germany; 2 Department of Medical Affairs, MSD Sharp & Dohme GmbH, Munich, Germany; 3 Center for Observational and Real-World Evidence (CORE), Merck & Co., Inc., Rahway, NJ USA; 4 EU Real World Evidence, Xcenda GmbH, Hannover, Germany; 5 Department for Children and Adolescents, Division for Neonatology, Main-Kinzig-Kliniken GmbH, Gelnhausen, Germany; 6 Department of Neonatology, University Children’s Hospital, Tuebingen, Germany; 7 Institute for Medical Virology and Epidemiology of Viral Diseases, University of Tuebingen, Tuebingen, Germany; 8 Section of Phoniatrics and Pediatric Audiology, Department of Otolaryngology, University Hospital Regensburg, Regensburg, Germany; 9 Institute of Medical Microbiology and Hygiene, University Hospital Regensburg, Regensburg, Germany; 10 Department of Health Economics and Health Care Management, Bielefeld School of Public Health, Bielefeld University, Bielefeld, Germany; 11 Global Medical and Scientific Affairs, MSD Sharp & Dohme GmbH, Munich, Germany

**Keywords:** Congenital CMV infection, Newborns, Health economic burden, Germany, Administrative data

## Abstract

**Background:**

Congenital cytomegalovirus (cCMV) infection can cause severe neurological damage, growth retardation, hearing loss, and microcephaly in infants. We aimed at assessing healthcare costs of infants with recorded cCMV diagnosis in an administrative claims database in the first 2 years of life.

**Methods:**

We conducted a retrospective, controlled cohort study using German claims data from the Institute for Applied Health Research Berlin (InGef) database. Incremental healthcare costs during the first and second year of life were assessed by matching (1:60) infants with cCMV diagnoses ≤ 90 days after birth (cCMV_90_ cohort) to infants without cCMV diagnosis (“representative” controls) and infants with cCMV diagnoses ≤ 21 days after birth plus specific symptoms (cCMV_21-S_) to infants without cCMV and any ICD-10-GM records (besides Z00-Z99) until 4^th^ preventive health check-up (“healthy” controls). Due to missing data, mean imputation was applied for aids and remedies costs.

**Results:**

We identified 54 and 24 infants born 2014–2018 for the cCMV_90_ and cCMV_21-S_ cohorts, respectively. During the first year, mean (median) healthcare costs were significantly higher in cCMV_90_ cases vs. “representative” controls (€22,737 (€9759) vs. €3091 (€863), p < 0.001), with 87.2% inpatient costs. Healthcare costs for cCMV_21-S_ cases compared to “healthy” controls were €34,498 (€20,924) vs. €680 (€569), p < 0.001. Differences decreased for both comparisons in the second year but remained statistically significant.

**Conclusions:**

cCMV comprises a considerable economic burden for the German healthcare system (€19,646 to €33,818 higher mean costs for infants with recorded cCMV diagnosis in the first year of life). Attempts should be made to reduce this burden.

**Supplementary Information:**

The online version contains supplementary material available at 10.1186/s12962-022-00411-x.

## Background

Cytomegalovirus (CMV) belongs to *Herpesviridae*, establishing lifelong persistence after primary infection. Seroprevalence in the general population is estimated to be 45–100% [1]. CMV infections can cause serious damage to the fetus when infected during pregnancy and has been suggested to be associated with permanent sequelae including neurological damage, hearing loss, and microcephaly [2, 3]. Congenital CMV (cCMV) infection has been reported to cause more congenital disabilities than Down syndrome, neural tube defect, or fetal alcohol syndrome [2, 4]. CMV-seronegative women with primary CMV infection during pregnancy have a considerably higher risk of giving birth to a symptomatically infected infant than CMV-seropositive women experiencing reactivation or reinfection during pregnancy [5]. In Germany, 46–60% of pregnant women are assumed to be seronegative [2, 6] and 0.2–0.5% among all living newborns are assumed to be congenitally infected [2]. About 12–20% of congenitally infected newborns are symptomatic at birth [7, 8], 40–58% of infants with symptomatic CMV infections suffer from permanent and late-onset sequelae like hearing loss and cognitive disabilities, and mortality in symptomatic infants with cCMV has been reported to be up to 5%, with single estimates reaching up to 10% [7].

For Germany, published data on the health economic burden of cCMV in infants are limited. A cost-of-illness study using data from 2008 [9] estimated lifetime average direct and indirect societal costs of cCMV to be €766,444, but costs were not directly compared to infants without cCMV. Another study from the Netherlands suggested that average healthcare costs of children with cCMV in the first 6 years of life are almost twice as high as healthcare costs of children without cCMV (€6113 vs. €3570) [8].

The present study covers one part of a large retrospective study that assessed the burden of cCMV in terms of cCMV sequelae, healthcare resource utilization and healthcare costs in infants during the first and second year of life from the perspective of the German Statutory Health Insurance (SHI). In this paper we present incremental healthcare costs of infants with cCMV compared to infants without cCMV. Due to challenges in retrospective identification of infants with cCMV [10], and special challenges concerning identification of cCMV in administrative claims databases, such as undercoding of cCMV [11–13], we developed two definitions for infants with cCMV. By comparing different definitions of cCMV and control cohorts, we aimed at providing a plausible span for cCMV-related healthcare costs in infants with recorded cCMV diagnosis from the perspective of the SHI in Germany.

## Methods

### Study design

We conducted a retrospective, controlled matched cohort analysis using German SHI claims data from 2014 to 2019 to assess the incremental healthcare costs of cCMV in infants with and without recorded cCMV diagnosis during their first and second year of life. The first and second year of life were defined as the first 1–365 and 366–730 days of life, respectively.

### Database

We utilized the Institute for Applied Health Research Berlin (InGef) database with SHI claims data from about 60 different health insurances covering approximately 8 million lives with a well-distributed geographic representation of the German population and good external validity in terms of morbidity, mortality, and drug use [14].

Claims data from the participating SHIs are joined in a specialized trust center, anonymized, and transferred to InGef before the data are made available for research. By German legislation, the analysis of claims data from the SHI is permitted and does not require the approval of an ethics committee.

### Study population

Infants in the InGef database born 2014–2018 were included in this study. Newborns needed to be continuously observable for at least 365 or 730 days of life, except for infants who deceased. Infants with pre-specified diagnoses of immunocompromising diseases (leukemia, human immunodeficiency virus (HIV), solid organ transplant, or stem cell transplant) during the first 365 days of life were excluded from the study population (see Additional file 1: Table S1).

Two cCMV cohorts and control groups were defined:

- Study cohorts
  - cCMV-cohort 1 (cCMV_90_): all infants with a documented diagnosis for cCMV (ICD-10-GM P35.1) during the first 90 days of life irrespective of documented clinical symptoms.
  - cCMV-cohort 2 (cCMV_21-S_): all infants with documented inpatient diagnosis for cCMV (ICD-10-GM P35.1) and at least one cCMV-specific symptom recorded during any hospital admission in the first 21 days of life (including birth).
- Control groups
  - Control group 1 (“representative”): all infants without a documented diagnosis for cCMV (ICD-10-GM P35.1) and CMV (ICD-10-GM B25) at any time during their individual observation period in the database.
  - Control group 2 (“healthy”): all infants from control group 1 with no ICD-10-GM records (except Z00–Z99 codes) in the quarters of preventive check-ups for children (until 4th preventive check-up).

A more detailed description of selection criteria including information on the German claims data coding system can be found in Additional file 1: Case definition.

A distinction should be made between congenital and postnatal CMV infection as the long-term complications and treatment options differ considerably [10]. CMV infections should be confirmed by laboratory diagnosis within the first 21 days of life using salvia and/or urine for diagnosis to be classified as congenital [15, 16]. Thereafter, postnatal CMV transmission by other infants, breastfeeding or body fluids is possible [5, 17]. Therefore, our cCMV_21-S_ cohort included infants with recorded inpatient cCMV diagnosis during the first 21 days of life in combination with at least one pre-defined cCMV-associated symptom/disease [3, 17], to strengthen accuracy. The cCMV_90_ cohort included infants with cCMV diagnoses during the first 90 days of life irrespective of symptom records, as during these 90 days, retrospective diagnosis of cCMV infection can be performed by polymerase chain reaction using dried blood spots (DBS, Guthrie card). Due to German data protection regulations, Guthrie cards must be destroyed after 3 months, making a reliable diagnosis of cCMV infection nearly impossible thereafter [10].

Here, we present the comparison of the cCMV_90_ cohort to ”representative” controls and the comparison of the cCMV_21-S_ cohort to a more artificial cohort of ”healthy” controls. The first comparison will produce the lowest and the latter comparison the highest increment estimate for healthcare costs concerning all four possible comparisons, providing a plausible span for cCMV-related healthcare costs of infants with recorded cCMV diagnosis from the perspective of the SHI in Germany. The two complementary comparisons (cCMV_90_ to “healthy” and cCMV_21-S_ to “representative”) are reported in Additional file 2.

### Outcomes

Healthcare costs were analyzed as costs for outpatient care, inpatient care, outpatient pharmaceuticals, remedies, devices and aids, and summarized as total costs. Outcomes were analyzed separately for the first and second year of life.

### Statistical analysis

We performed a 1:60 direct matching. The matching ratio selected was data-driven based on the highest number of matches available for all group comparisons. Matching was based on gender, year-specific quarter of birth, and observability of the newborns.

Descriptive analyses were performed for patient demographics, and healthcare costs in terms of mean values, medians, ranges, and standard deviations for continuous variables and absolute and relative frequencies for categorical variables.

As data for remedies, devices and aids are not completely available for all individuals in the database due to technical issues with data transfers, single imputation was applied using the mean costs of infants with available data.

Statistical significance tests of descriptive differences comprised non-parametric Mantel–Haenszel matched-pairs analysis for dichotomous variables and Wilcoxon rank-sum tests for continuous variables. P < 0.05 was considered as statistically significant. Additionally, for continuous variables, mean differences with 95% confidence intervals (CI) were calculated.

To assess the effect of potential cost outliers, the cost analysis was repeated using 95% one-sided upper winsorization, i.e., infants with cost values above 95% percentile were not excluded, but their costs were replaced with their group’s 95% percentile cost value.

## Results

### Study population

**Fig. 1:**
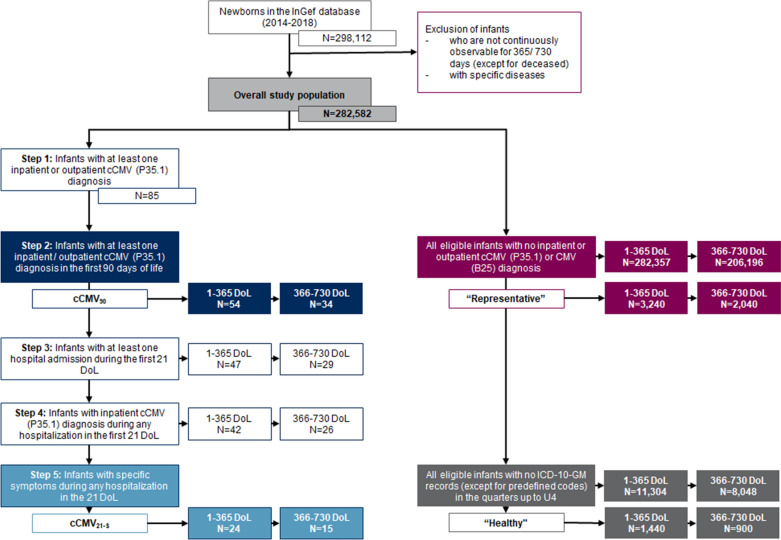
Patient selection process. *InGef* Institute for Applied Health Research Berlin, *cCMV* congenital cytomegalovirus, *CMV* cytomegalovirus, *cCMV*_*90*_ infants with cCMV diagnosis during the first 90 days of life, *“Representative”* infants with no cCMV or CMV diagnosis in the observation period; cCMV_21-S_, infants with cCMV diagnosis and symptoms during the first 21 days of life, *“Healthy”* infants with no ICD-10-GM diagnosis (except Z-diagnoses) until 4th preventive health checkup and no cCMV or CMV diagnosis in the observation period, *DoL* days of life, *ICD-10-GM* International Statistical Classification of Diseases and Related Health Problems, 10th Revision, German Modification, *U4* fourth preventive checkup examination

Overall, N = 282,582 infants born between 2014 and 2018, who fulfilled the inclusion criteria, were available in the InGef database (Fig. 1). N = 54 infants (55.6% males) fulfilled the cCMV_90_ definition. Of those, N = 34 were observable in the second year of life. In the cCMV_21-S_ cohort, this applied to N = 24 (62.5% males) and N = 15 infants, respectively, (Fig. 1). Around 30.0% of newborns in the cCMV_90_ cohort and 50.0% in the cCMV_21-S_ cohort had low birth weight (< 2500 g), whereas > 95% in both control groups showed regular birth weight (Table 1).

**Table 1:** Baseline demographics and clinical characteristics^a^ after matching

Characteristic	cCMV_90_ cohort N (%)	“Representative” controls N (%)	cCMV_21-S_ cohort N (%)	“Healthy” controls N (%)
Observability				
1–365 days of life	54 (100.0)	3240 (100.0)	24 (100.0)	1440 (100.0)
366–730 days of life	34 (63.0)	2040 (63.0)	15 (62.5)	900 (62.5)
Gender				
Male	30 (55.6)	1,800 (55.6)	15 (62.5)	900 (62.5)
Female	24 (44.4)	1,440 (44.4)	9 (37.5)	540 (37.5)
Birth quarter				
Q1	11 (20.4)	660 (20.4)	6 (25.0)	360 (25.0)
Q2	14 (25.9)	840 (25.9)	7 (29.2)	420 (29.2)
Q3	14 (25.9)	840 (25.9)	< 5 (-)	180 (12.5)
Q4	15 (27.8)	900 (27.8)	8 (33.3)	480 (33.3)
Birth weight				
Extremely low (P07.0–)	< 5 (–)	11 (0.3)	< 5 (–)	0 (0.0)
Low (P07.1–)	14 (25.9)	128 (4.0)	12 (50.0)	0 (0.0)
High (P08.1 or P08.2)	0 (0.0)	19 (0.6)	0 (0.0)	0 (0.0)
Normal (infants with none of the stated codes)	37 (68.5)	3082 (95.1)	9 (37.5)	1440 (100.0)
Region				
North^b^	10 (18.5)	574 (17.7)	< 5 (–)	240 (16.7)
East^c^	9 (16.7)	343 (10.6)	7 (29.2)	119 (8.3)
West^d^	21 (38.9)	1171 (36.1)	8 (33.3)	520 (36.1)
South^e^	14 (25.9)	1145 (35.3)	7 (29.2)	560 (38.9)
Unknown	0 (0.0)	7 (0.2)	0 (0.0)	< 5 (–)

*cCMV* congenital cytomegalovirus, *cCMV*_*90*_ infants with cCMV, diagnosis during the first 90 days of life, *“Representative”*, infants with no cCMV or CMV diagnosis in the observation period; *cCMV*_*21-S*_ infants with cCMV diagnosis and symptoms during the first 21 days of life, *“Healthy”* infants with no ICD-10-GM diagnosis (except Z-diagnoses) until 4th preventive health checkup and no cCMV or CMV diagnosis in the observation period; *Q1* January 1st–March 31st, *Q2* April 1st–June 30th, *Q3* July 1st–September 30th, *Q4* October 1st–December 31st

^a^Baseline demographics and clinical characteristics were assessed during the first 1–365 days of life

^b^North includes federal states Schleswig–Holstein, Hamburg, Bremen, Lower Saxony, and Mecklenburg-Western Pomerania

^c^East includes federal states Thuringia, Brandenburg, Berlin, Saxony, and Saxony-Anhalt

^d^West includes federal states North Rhine-Westphalia, Saarland, Rhineland-Palatinate, and Hesse

^e^South includes federal states Bavaria and Baden-Wuerttemberg

### Healthcare costs

**Fig. 2:**
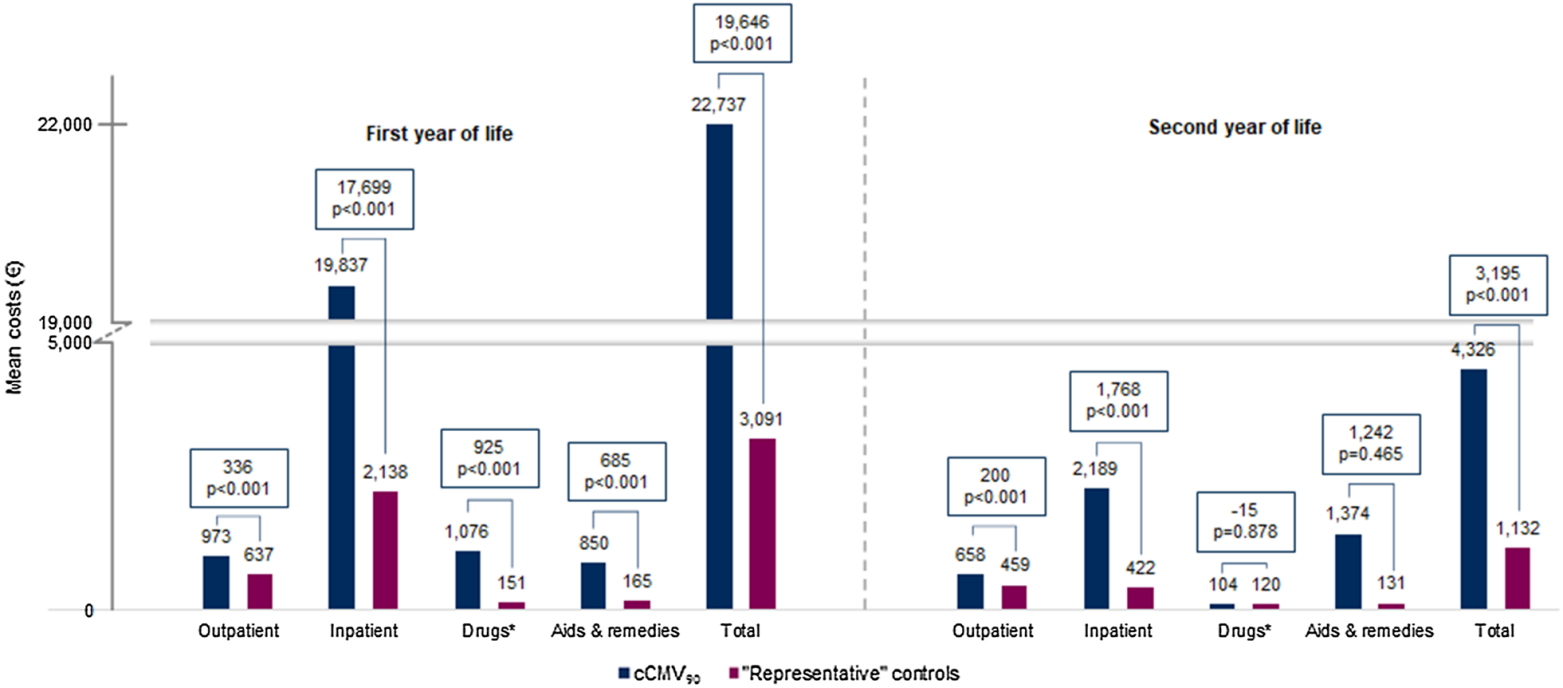
Mean healthcare costs in first and second year of life—cCMV_90_ versus "representative" controls. (*) Drugs include costs of outpatient pharmaceuticals; costs of inpatient pharmaceuticals are included in inpatient costs. Incremental costs are shown with respective p-values. P-value < 0.05 was considered as statistically significant (Wilcoxon rank-sum test). Figures were commercially rounded, which may result in minor calculation differences. *cCMV* congenital cytomegalovirus, *cCMV*_*90*_ infants with cCMV diagnosis during the first 90 days of life, *“Representative”* infants with no cCMV or CMV diagnosis in the observation period

**Fig. 3:**
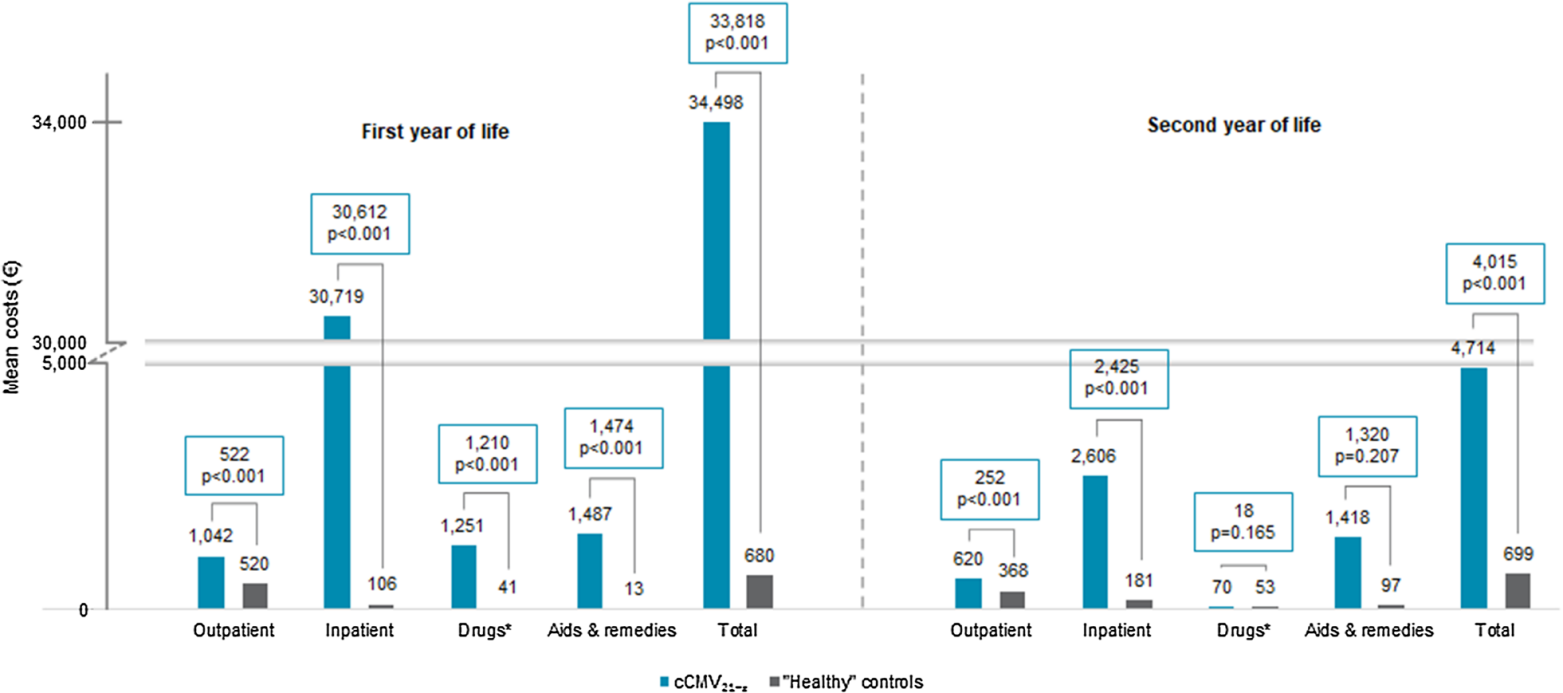
Mean healthcare costs in first and second year of life—cCMV_21-S_ versus "healthy" controls. (*) Drugs include costs of outpatient pharmaceuticals; costs of inpatient pharmaceuticals are included in inpatient costs. Incremental costs are shown with respective p-values. P-value < 0.05 was considered as statistically significant (Wilcoxon rank-sum test). Figures were commercially rounded, which may result in minor calculation differences. *cCMV* congenital cytomegalovirus, *cCMV*_*21-S*_ infants with inpatient cCMV diagnosis and symptoms during the first 21 days of life, *“Healthy”* infants with no ICD-10-GM diagnosis (except Z-diagnoses) until 4th preventive health checkup (U4) and no cCMV or CMV diagnosis in the observation period

Both cCMV cohorts had statistically significant higher total healthcare costs per infant compared to their respective control groups during the first two years of life (Figs. 2, 3). In the first year of life, total mean (median) healthcare costs per infant were about seven times higher in cCMV_90_ cohort compared to the “representative” controls (€22,737 (€9759) vs. €3091 (€863), p < 0.001) with a mean difference of €19,646 (CI €9814–€29,477). For the cCMV_21-S_ cohort, mean (median) total costs per infant were about 51 times higher compared to “healthy” controls (€34,498 (€20,924) vs. €680 (€569), p < 0.001) resulting in a mean difference of €33,818 per infant (CI €15,811–€51,825) during the first year of life. Costs for inpatient care accounted for 87.2% (cCMV_90_) and 89.0% (cCMV_21-S_) of total mean costs and were thus the main cost driver during the first year of life in both cCMV cohorts (Table 2).

**Table 2:** All-cause healthcare costs^a^ during the first 1–365 days of life

Cost domain	cCMV_90_ cohort	“Representative” controls	Mean difference (CI)	p-value^b^	cCMV_21-S_ cohort	“Healthy” controls	Mean difference (CI)	p-value^b^
N (infants)	54	3240			24	1440		
Outpatient								
Sum	52,563	2,065,429			25,007	749,019		
Mean	973	637	336 (234–438)	< 0.01	1042	520	522 (348–696)	< 0.01
SD	382	281			435	122		
Min	328	0			328	214		
Q1	695	505			818	440		
Median	947	604			1009	516		
Q3	1171	721			1244	589		
Max	2082	6262			2082	1369		
Inpatient								
Sum	1,071,210	6,927,803			737,247	153,331		
Mean	19,837	2138	17,699 (8477–26,921)	< 0.01	30,719	106	30,612 (13,639–47,586)	< 0.01
SD	34,555	9217			42,425	694		
Min	0	0			3238	0		
Q1	3,683	0			10,531	0		
Median	8,343	0			14,723	0		
Q3	19,407	986			29,972	0		
Max	194,846	202,112			194,846	14,618		
Pharmaceuticals								
Sum	58,093	487,828			30,013	58,617		
Mean	1076	151	925 (456–1395)	< 0.01	1251	41	1210 (528–1892)	< 0.01
SD	1757	707			1705	57		
Min	1	0			6	0		
Q1	71	29			85	15		
Median	154	54			503	29		
Q3	1184	103			1434	49		
Max	7272	22,369			5903	1584		
Aids and remedies^c^								
Sum	45,924	534,654			35,681	18,142		
Mean	850	165	685 (175–1195)	< 0.01	1487	13	1474 (384–2565)	< 0.01
SD	1910	655			2726	55		
Min	0	0			0	0		
Q1	0	0			0	0		
Median	208	29			394	0		
Q3	850	165			1487	13		
Max	10,694	17,737			10,694	757		
Total								
Sum	1,227,790	10,015,714			827,948	979,109		
Mean	22,737	3091	19,646 (9814–29,477)	< 0.01	34,498	680	33,818 (15,811–51,825)	< 0.01
SD	36,838	9935			45,008	735		
Min	655	0			5015	214		
Q1	4958	653			12,950	479		
Median	9759	863			20,924	569		
Q3	21,623	2106			31,201	667		
Max	212,552	211,210			212,552	15,662		

*cCMV* congenital cytomegalovirus, *cCMV*_*90*_ infants with cCMV diagnosis during the first 90 days of life, *“Representative”* infants with no cCMV or CMV diagnosis in the observation period, *cCMV*_*21-S*_ infants with inpatient cCMV diagnosis and symptoms during the first 21 days of life, *“Healthy”* infants with no ICD-10-GM diagnosis (except Z-diagnoses) until 4th preventive health checkup and no cCMV or CMV diagnosis in the observation period, *CI* 95% confidence interval, *SD* standard deviation, *Min* minimum; *Q1*, 25th percentile; *Q3*, 75th percentile, *Max* maximum

^a^Costs are displayed in Euros (€). Figures were commercially rounded, which may result in minor calculation differences

^b^P-value < 0.05 was considered as statistically significant (Wilcoxon rank-sum test)

^c^Data for aids and remedies were not completely available for all individuals in the database (18.5% cCMV_90_ cohort and 31.6% respective controls, 12.5% cCMV_21-S_ cohort and 25.9% respective controls), and single imputation was applied using the mean costs of infants with available data

In the second year of life, cost differences decreased between both cCMV cohorts and their respective controls but remained statistically significant. Total mean (median) healthcare costs per infant were more than three times higher in cCMV_90_ cohort compared to the “representative” controls (€4326 (€1464) vs. €1132 (€562), p < 0.001) with mean difference of €3195 (CI €546–€5844). For the cCMV_21-S_ cohort, mean (median) total costs per infant were more than six times higher compared to “healthy” controls (€4714 (€1977) vs. €699 (€441), p < 0.001), leading to a mean difference of €4015 (CI € − 1091 to €9121) during the second year of life. Costs for inpatient care remained the main cost driver in the second year of life with more than 50% of total healthcare costs occurring in the inpatient setting (Table 3).

**Table 3:** All-cause healthcare costs^a^ during the first 366–730 days of life

Cost domain	cCMV_90_ cohort	“Representative” controls	Mean difference (CI)	p-value^b^	cCMV_21-S_ cohort	“Healthy” controls	Mean difference (CI)	p-value^b^
N (infants)	34	2040			15	900		
Outpatient								
Sum	22,386	935,556			9295	331,279		
Mean	658	459	200 (75–325)	< 0.01	620	368	252 (124–379)	< 0.01
SD	369	351			251	179		
Min	235	0			235	0		
Q1	400	313			470	265		
Median	622	414			654	350		
Q3	792	533			774	444		
Max	2179	9199			1100	2186		
Inpatient								
Sum	74,440	860,412			39,097	163,295		
Mean	2189	422	1768 (− 114 to 3650)	< 0.01	2606	181	2425 (− 1473 to 6323)	< 0.01
SD	5580	3509			7702	724		
Min	0	0			0	0		
Q1	0	0			0	0		
Median	452	0			485	0		
Q3	1841	0			1325	0		
Max	30,335	144,020			30,335	7505		
Pharmaceuticals								
Sum	3549	244,180			1052	47,250		
Mean	104	120	− 15 (− 74 to 43)	0.88	70	53	18 (− 19 to 55)	0.17
SD	146	741			73	78		
Min	0	0			0	0		
Q1	27	24			25	11		
Median	50	51			40	32		
Q3	95	101			92	66		
Max	654	28,763			260	1199		
Aids and remedies^c^								
Sum	46,714	268,237			21,266	87604		
Mean	1374	131	1242 (232–2253)	0.46	1418	97	1320 (− 97 to 2738)	0.21
SD	3005	988			2794	1576		
Min	0	0			0	0		
Q1	0	0			0	0		
Median	0	0			0	0		
Q3	1374	131			1418	97		
Max	12,669	27,166			9002	47,207		
Total								
Sum	147,089	2,308,385			70,711	629,428		
Mean	4326	1132	3195 (546–5844)	< 0.01	4714	699	4015 (− 1091 to 9121)	< 0.01
SD	7860	4432			10,087	1904		
Min	253	0			253	7		
Q1	618	404			993	323		
Median	1464	562			1977	441		
Q3	3728	809			2537	605		
Max	40,384	159,540			40,384	52,518		

*cCMV* congenital cytomegalovirus, *cCMV*_*90*_ infants with cCMV diagnosis during the first 90 days of life, *“Representative”* infants with no cCMV or CMV diagnosis in the observation period, *cCMV*_*21-S*_ infants with inpatient cCMV diagnosis and symptoms during the first 21 days of life, *“Healthy”* infants with no ICD-10-GM diagnosis (except Z-diagnoses) until 4th preventive health checkup and no cCMV or CMV diagnosis in the observation period, *CI* 95% confidence interval, *SD* standard deviation, *Min* minimum, *Q1* 25th percentile, *Q3* 75th percentile, *Max* maximum

^a^Costs are displayed in Euros (€). Figures were commercially rounded, which may result in minor calculation differences

^b^P-value < 0.05 was considered as statistically significant (Wilcoxon rank-sum test)

^c^Data for aids and remedies were not completely available for all individuals in the database (20.6% cCMV_90_ cohort and 34.0% respective controls, 20.0% cCMV_21-S_ cohort and 33.0% respective controls), and single imputation was applied using the mean costs of infants with available data

## Discussion

This is the first study investigating healthcare costs in infants with recorded cCMV diagnoses during their first and second year of life for Germany using SHI claims data. Our study highlights a significantly higher burden in infants with recorded cCMV diagnosis in terms of healthcare costs compared to infants without cCMV diagnosis, suggesting that on average, one infant with recorded cCMV diagnosis produced €19,646–€33,818 more costs for the SHI during their first life year compared to one infant without cCMV diagnosis. Inpatient costs were the main cost driver. The study cohort had information on 54 infants with recorded cCMV diagnoses out of 282,582 births, which amounts to a birth prevalence of 0.02%. As other sources suggest that the actual birth prevalence of cCMV in Germany may be between 0.2% and 0.5% [2], this gap may indicate considerable underdiagnosis in current practice [13]. Therefore, the derived cost estimates might only be the tip of the iceberg, leaving a larger dark figure of children with cCMV, potentially due to less severe symptoms or misdiagnosed etiology. For the interpretation of the results of our study this implies that the cost estimates for the identified infants with recorded cCMV diagnosis in the utilized claims database provide only limited transferability for the complete population of infants with cCMV in Germany. As our study population included only a fraction of all infants with cCMV in Germany, it is reasonable to assume that the overall direct costs for the SHI in Germany are probably higher. At the same time, it is noteworthy that those infants with cCMV which we did not cover in our study likely were a mixture of asymptomatic and mis- or undiagnosed symptomatic infants. Whereas the latter might be as cost-intensive as the infants we captured with our study, the fraction of asymptomatic infants is presumably less cost-intensive. As a result, we expect that mean costs per infant in the complete population of infants with cCMV would be lower. Since no further literature for Germany covering this research question is currently available, our study provides a relevant first approximation of healthcare costs for the SHI in Germany for infants identifiable in administrative data despite these remaining uncertainties.

Also internationally, only limited published data assessing comparable research questions are available. A claims data analysis from the US found that costs of infants with cCMV diagnoses during the first year of life were on average seven times higher compared to matched infants without cCMV diagnosis [18]. When comparing our cCMV_90_ cohort with “representative” controls, we found differences of similar magnitudes. However, the US study included infants with cCMV diagnosis performed during the complete first year of life [18], whereas our cCMV_90_ cohort comprised only infants with cCMV diagnosis during the first 90 days of life. A nationwide retrospective cohort study from the Netherlands found almost two times higher mean healthcare costs per child in the first 6 years of life in 156 infants with cCMV-infection confirmed by screening of a retrospectively recruited sample of children’s of DBS (average difference: €2544) compared to a matched cCMV-negative control group [8]. However, contrary to our results, the difference was statistically not significant. Further, the comparability between the study from the Netherlands and our results is limited due to different underlying study populations (targeted diagnostics in Germany that were most probably triggered by clinical symptoms vs. a broader retrospective sample screening in the Netherlands which allowed for capturing asymptomatic children) and time periods (first two vs. first six years of life). Further studies estimated the lifetime costs of cCMV [9, 19], costs of hospitalizations related to cCMV in < 1-year-old infants [20] or provided a statistic cost model for cCMV for individuals of all ages [21]. A retrospective cohort study from Israel described direct healthcare costs in infants with cCMV compared to controls during the first 4 years of life [22]. They also found highest incremental healthcare costs during the first year of life.

Depending on the inclusion criteria for the cCMV cohorts and the respective control group definitions, the magnitude of group differences varies, with generally larger group differences found for the comparison of the cCMV_21-S_ cohort with “healthy” controls. These differences in magnitude of results were expected, as the cCMV_21-S_ cohort comprised the sub-group of presumably severe cCMV cases already symptomatic at birth, whereas the cCMV_90_ cohort allowed for including cases where congenital infection could still reasonably be assumed despite a potentially later symptom onset. With these two definitions we aimed at building a range for the actual healthcare costs in infants with cCMV. The definitions of the two control groups analogously followed this intention. The comparison of the cCMV_21-S_ cohort with “healthy” controls should represent the most extreme expected differences (comparing the cCMV worst case scenario with the best-case control group scenario), whereas the comparison of the cCMV_90_ cohort with “representative” controls may provide more conservative incremental cost estimates. Complementary comparisons of the cCMV_90_ cohort with “healthy” controls and the cCMV_21-S_ cohort with “representative” controls resulted in point estimates between the extremes described above, which may be closer to a population average (see Additional file 2).

As noted in the case definition, even though infants in our cCMV_90_ cohort were not required to have documentation of any specific symptom, we assume that all infants with a cCMV record during the first 90 days of life may have been symptomatic to some extent, as no universal newborn screening for cCMV exists in Germany. Therefore, we hypothesize that these infants must have been detected and diagnosed after targeted investigation, possibly triggered by signs or symptoms in their development. As physicians may code only ICD-10-GM codes that are relevant for reimbursement purposes, it is possible that not all symptoms were recorded.

Since clinical or laboratory data is not available in the database, it is impossible to verify if the recorded cCMV diagnoses were based on virological results. Consequently, the classification of infants in the cCMV cohorts depends to some extent on the ICD coding behavior of physicians. To face these limitations, we established a detailed identification process for cCMV infants. Furthermore, reliable diagnosis of cCMV depends on direct virus detection either during the first 14 days post-partum using polymerase chain reaction diagnosis or from day 1–21 post-partum using virus culture [15, 16]. Additionally, cCMV may be retrospectively diagnosed until school age using DBS from newborn screening [23]. As DBS are destroyed in Germany 90 days after birth, the cCMV_90_ definition reflects plausible time frames of potential virological diagnosis of cCMV. Since the utilized database does not provide any details on virological cCMV diagnosis, we cannot fully exclude that the cCMV_90_ cohort may comprise cases of postnatally acquired CMV (miscoded as cCMV). Therefore, the cCMV_21-S_ cohort might provide a higher probability of valid cCMV diagnoses but misses out on infants with symptoms detected only after the first 21 days of life.

In sum, our study population presumably consisted of infants which were symptomatic in their first 21 and likely in their first 90 days of life. Assumptions on costs of all infants with cCMV, including infants with asymptomatic cCMV or misdiagnosed symptomatic infants, cannot be made in this study, as these are likely not covered by our study population. Consequently, the cost estimates cannot be extrapolated to the overall population of infants with cCMV in Germany, as this would probably overestimate the costs per infants with cCMV for the SHI. As we only assessed the first 2 years of life, costs thereafter as well as costs of infants with cCMV but a later symptom onset (i.e., after the first 90 days of life) were not covered. However, we may have underestimated the total costs for symptomatic infants as certain disability support measures, like special kindergartens, are incompletely represented in the InGef database and might additionally be requested more often for children after the age of 2 years. The representativeness of cost estimates from administrative databases for the overall cCMV population has also been questioned elsewhere [12, 24]. Even though the utilization of claims databases is associated with limitations, they still provide valuable insights into the healthcare costs of infants with recorded cCMV diagnosis, especially considering the current scarcity of data in this field.

As valid data for remedies, devices, and aids were not available for all infants, mean imputation was applied for infants with invalid data. By using the mean for imputation of missing remedies, devices, and aids data, the results for this cost domain and consequently the total costs are possibly skewed as the mean could be influenced by outliers. As the main message of this study refers to mean values, we decided to use the mean imputation for reasons of consistency.

The small sample sizes of cCMV cohorts also bared the danger of skewed data due to outliers. To adjust for potential outliers due to high-cost diseases (HIV, leukemia etc.) those were excluded from the study population. Additionally, cost results were winsorized at 95^th^ percentile as sensitivity analysis to assess the effect of potential cost outliers. Even after exclusion of high-cost diseases and winsorization, the results show a high variability of values indicating that healthcare cost in infants with cCMV infants may differ considerably (Additional files 1, 2).

Another limitation of the database and the study design is that the presented data can only to some extent provide detailed information on specific treatments. For instance, pharmaceuticals dispensed in the inpatient setting (e.g., valganciclovir) cannot be assessed separately as these costs are usually included in the compensation schemes for diagnosis related groups (DRG). However, this also reflects a major strength of this study as it shows the overall costs and resource utilization that occurred from the perspective of SHI. By comparing respective increments between infants with cCMV and infants without cCMV, we were able to identify the cost differences which are assumed to be related to cCMV.

## Conclusion

The results of this study underline the significant health economic burden in infants with recorded cCMV diagnosis for the SHI system in Germany and indicate the need for preventive steps and a comprehensive monitoring of cCMV infection and related sequelae. With on average €19,646–€33,818 higher annual costs during the first and €3195–€4015 higher annual costs during the second year of life for infants with recorded cCMV diagnosis compared to infants without cCMV, the currently measurable additional healthcare costs per infant with recorded CMV diagnosis are substantial. Even though extrapolation of the reported per case costs to the complete dark figure of infants with cCMV in Germany would likely overestimate the health economic impact of cCMV for the SHI in Germany, a considerable budget impact remains conceivable. An important question is which measures may be helpful to reduce this significant burden of cCMV in the future. As vaccines against CMV are not available, other steps for prevention of cCMV infection need to be implemented.

Systematic collection of information on cCMV infection in pregnant women and newborns could not only help raise awareness of the risks of cCMV, but also provide better estimates on the actual burden of disease. Without reliable numbers of cCMV infections, which could be provided by neonatal screening, projection of the real overall health economic burden for all infants with cCMV to national level—including days parents are absent from the job, lifelong support in case of disability etc.—remains incomplete.

Due to the focus on the first two life years, our results present only a small fraction of the actual health economic burden of cCMV for the SHI for infants with recorded cCMV diagnosis and do not consider any indirect or societal costs. Further research should assess societal and lifetime costs of cCMV to understand the complete picture and attempts should be made to reduce the burden of cCMV.

## Supplementary Information

**Additional file 1: Table S1**. Exclusion Criteria for Overall Study Population.** Table S2**. cCMV-specific Symptoms and Sequelae.** Table S3**. All-cause Healthcare Costsa During the First 1-365 Days of Life After Winsorization.** Table S4**. All-cause Healthcare Costsa During the First 366-730 Days of Life After Winsorization

**Additional file 2. Table S1**. Baseline Demographic and Clinical Characteristicsa After Matching–Complementary Comparisons.** Table S2.** All-cause Healthcare Costsa During the First 1-365 Days of Life, Unadjusted–Complementary Comparisons.** Table S3**. All-cause Healthcare Costsa During the First 366-730 Days of Life, Unadjusted – Complementary Comparisons.** Table S4**. All-cause Healthcare Costsa During the First 1-365 Days of Life After Winsorization – Complementary Comparisons.** Table S5**. All-cause Healthcare Costsa During the First 366-730 Days of Life After Winsorization–Complementary Comparisons.

## Data Availability

The data used in this study was retrieved from the Institute for Applied Health Research Berlin (InGef) Research Database (www.ingef.de) and cannot be made available in the manuscript, the supplemental files, or in a public repository due to German data protection laws (Bundesdatenschutzgesetz). To facilitate the replication of results, anonymized data used for this study are stored on a secure drive at the Institute for Applied Health Research Berlin (InGef) GmbH. Access to the data used in this study can only be provided to external parties under the conditions of the cooperation contract of this research project and can be assessed upon request, after written approval at InGef GmbH (Tel. + 49 (30) 21 23 36-471; info@ingef.de), if required.

## Abbreviations

CI: Confidence interval
cCMV: Congenital cytomegalovirus
CMV: Cytomegalovirus
DBS: Dried blood spots (Guthrie Card)
DRG: Diagnosis related groups
HIV: Human immunodeficiency virus
ICD-10-GM: International Classification of Diseases, 10th Revision, German Modification
InGef: Institut für angewandte Gesundheitswissenschaften Berlin (Institute for Applied Health Research Berlin)
SHI: Statutory Health Insurance
