# Supplementary material for: Healthcare costs of congenital cytomegalovirus (cCMV) disease in infants during the first two years of life: a retrospective German claims database analysis
Source: Cost Eff Resour Alloc. 2023 Jan 23;21:8. doi: 10.1186/s12962-022-00411-x (PMC9872342; doi:10.1186/s12962-022-00411-x)
Supplement: Supplementary file 1 — Additional file 1: Table S1. Exclusion Criteria for Overall Study Population. Table S2. cCMV-specific Symptoms and Sequelae. Table S3. All-cause Healthcare Costsa During the First 1-365 Days of Life After Winsorization. Table S4. All-cause Healthcare Costsa During the First 366-730 Days of Life After Winsorization [file 12962_2022_411_MOESM1_ESM.docx]

**Additional file 1**

***Supplementary Material: Case Definition***

All newborns in the Institute for Applied Health Research Berlin (InGef) Database from 2014-2018 were considered for this analysis. For the assessment of the first year of life (*second year of life*) infants born in 2014-2018 (*born in 2014-2017*), needed to be continuously observable for at least 365 days of life (*730 days of life*) including birth date, except for infants who deceased in the follow-up period. For the outcome analyses of the identified infants, claims data from 2014-2019 was analyzed.

From all identified newborns, infants with International Statistical Classification of Diseases and Related Health Problems, 10th Revision, German Modification (ICD-10-GM) records of defined, immunocompromised diseases, which may increase the risk of postnatal CMV infection (see Supplementary Table 1) were excluded.

All remaining infants served as the overall study population from which the congenital cytomegalovirus (cCMV) cohorts as well as the two control groups were drawn.

The patient selection process is described in detail in the following.

***cCMV Cohorts***

From the identified newborns, two cohorts of infants with cCMV were identified. The following steps were applied to identify infants in cCMV-cohort 1 (cCMV_90_):

**Step 1**: all infants with an ICD-10-GM diagnosis record for cCMV (P35.1) in the inpatient sector (primary or secondary diagnosis) or outpatient sector (verified or suspected diagnosis) in the first 365 days were identified.

**Step 2**: of these, all infants were identified if they had

1. an inpatient diagnosis (primary or secondary diagnosis) for cCMV (P35.1) during any hospital stay that began in the first 90 days of life (including birth date).

***or***

1. an outpatient diagnosis (verified or suspected diagnosis) for cCMV (P35.1) within the first 90 days of life. As outpatient diagnoses in Germany are only available on a quarterly basis, the day-specific records of Official German Remuneration Scheme for Outpatient Care (Einheitlicher Bewertungsmaßstab, EBM) codes were used as a proxy to estimate the date of the outpatient diagnosis. If an EBM code was recorded within the 90-days window by the same physician who had recorded the verified or suspected cCMV diagnosis, the cCMV diagnosis was assumed to be recorded within the 90 days. The EBM code und the ICD-10-GM code were linked by the physician’s lifelong identification number (Lebenslange Arztnummer, LNAR).

To increase validity of outpatient diagnoses recorded within the 90 days window, the included outpatient (verified and suspected) diagnoses were only considered if they could be validated with a second cCMV diagnosis in the follow-up period. To this end, the infants were only further included if they had at least another inpatient (primary or secondary diagnosis) or at least one more outpatient (verified diagnosis only) cCMV diagnosis in another quarter or by another physician after the first (initial) outpatient diagnosis.

- The identified infants with an ICD-10-GM record for cCMV (P35.1) during an inpatient stay that began during the first 90 days of life (including birth) or with an outpatient diagnosis that could be linked to the first 90 days of life and validated with a second diagnosis were defined as cCMV cohort 1 (cCMV_90_)

Further steps were applied to identify infants in cCMV-cohort 2 (cCMV_21-S_):

**Step 3**: all infants with a hospital admission during the first 21 days of life (including birth) were identified.

**Step 4**: of these, all infants with an inpatient diagnosis (primary or secondary diagnosis) for cCMV (P35.1) during any hospital admission in this 21-day window were identified.

**Step 5**: of these, all infants with an inpatient diagnosis (primary or secondary diagnosis) of at least one pre-defined cCMV-specific symptom (Supplementary Table 2) during any hospital admission in this 21-day window were identified.

- The identified infants with cCMV diagnosis and specific symptoms during a hospital admission in their first 21 days of life (including birth) were defined as cCMV cohort 2 (cCMV_21-S_).

For the identification of cCMV we used ICD-10-GM codes, which is the official classification for the encoding of diagnoses in inpatient and outpatient medical care in Germany since 2000. Clinicians in the outpatient setting are required to add one of the following specifications to the ICD-10-GM codes: “suspected diagnosis”, “diagnosis ruled out”, “condition post diagnosis”, or “verified diagnosis”. For instance, “suspected” may be coded if the physician is not certain about the presence of the coded disease and a confirming laboratory analysis is still pending. To ensure the accuracy of cCMV diagnoses, only verified diagnoses and suspected diagnoses in the respective time frames were included. Outpatient verified and suspected diagnoses were only considered if they were verified with an additional inpatient (primary or secondary) cCMV diagnosis or at least one more outpatient (verified diagnosis only) cCMV diagnosis in another quarter or by another physician after the first (initial) outpatient diagnosis.

Note: Even though infants in our cCMV_90_ cohort were not required to have documentation of any specific symptom, we assume that all infants with a cCMV record during the first 90 days of life may have been symptomatic to some extent, as no universal newborn screening for cCMV exists in Germany. Therefore, we hypothesize that these infants must have been detected and diagnosed after targeted investigation, possibly triggered by signs or symptoms in their development. As physicians may code only ICD-10-GM codes that are relevant for reimbursement purposes, it is possible that not all symptoms were recorded.

**Control Groups**

From the overall study population, all infants with an outpatient diagnosis (verified diagnosis) or an inpatient diagnosis (primary or secondary diagnosis) for cCMV (P35.1) or CMV (B25) at any time in their observation period were excluded. Infants with B25 diagnosis were excluded to decrease the probability of including infants with cCMV in the control groups that might have been misdiagnosed with (postnatal) CMV. Out of the remaining infants, the following criteria defined eligibility for inclusion in the respective control groups:

- Control group 1 (“representative” controls): all remaining infants from the overall study population without cCMV (P35.1) or CMV (B25) ICD-10-GM codes
- Control group 2 (“healthy” controls): from control group 1, all infants who fulfilled the following inclusion criteria:
  - Infants with an outpatient record for 3^rd^ and 4^th^ preventive checkups^[[1]](#footnote-2)^ (U3 and U4) in the first 365 days of life were identified i.e., all infants with no record for U3 (EBM code 01713) AND/OR U4 (EBM code 01714) in the first 365 days were excluded. As the 2^nd^ checkup (U2, EBM code 01712) can generally also be conducted during an inpatient stay, infants were not excluded if they had no outpatient record for U2, but all infants were required to have an outpatient record for U3 and U4.
  - Each infants’ individual quarter of U4 was identified and served as the upper limit for the diagnosis free period for these infants. In the timeframe beginning from birth up to and including the individual quarter of U4 (the diagnosis free period), infants were not allowed to have any ICD-10-GM diagnosis records, except for predefined ICD-10-GM codes (Z00-Z99) in the inpatient sector (main or secondary diagnosis) or in the outpatient sector (verified diagnosis).

As controls needed to be continuously observable for at least as long as their matched cCMV cases, controls who deceased before the cases were excluded.

Table S1. Exclusion Criteria for Overall Study Population

| Type | Code | German Description | Group |
| --- | --- | --- | --- |
| ICD-10-GM | B20 | Infectious and parasitic diseases due to HIV disease [human immunodeficiency virus disease] | HIV |
| ICD-10-GM | B21 | Malignant neoplasms as a result of HIV disease [human immunodeficiency virus disease] | HIV |
| ICD-10-GM | B22 | Other specified diseases due to HIV disease [human immunodeficiency virus disease] | HIV |
| ICD-10-GM | B23 | Other disease states as a result of HIV disease [human immunodeficiency virus disease] | HIV |
| ICD-10-GM | B24 | Unspecified HIV disease [human immunodeficiency virus disease] | HIV |
| ICD-10-GM | Z21 | Asymptomatic HIV infection [human immunodeficiency virus infection] | HIV |
| ICD-10-GM | Z94 | Condition after organ or tissue transplantation | Transplantation |
| ICD-10-GM | T86 | Failure and rejection of transplanted organs and tissues | Transplantation |
| ICD-10-GM | U55 | Registration for organ transplantation | Transplantation |
| ICD-10-GM | Z75.6 | Registration for organ transplantation without urgency level HU (High Urgency) | Transplantation |
| ICD-10-GM | Z75.7 | Registration for organ transplantation with urgency level HU (High Urgency) | Transplantation |
| OPS | 5-335 | Lung transplant | Transplantation |
| OPS | 5-375 | Heart and heart-lung transplant | Transplantation |
| OPS | 5-504 | Liver transplant | Transplantation |
| OPS | 5-555 | Kidney transplant | Transplantation |
| OPS | 1-920.2 | Complete evaluation, with the inclusion of a patient on a waiting list for organ transplantation | Transplantation |
| OPS | 1-920.3 | Complete evaluation, with the inclusion or retention of a patient on a waiting list for organ transplantation | Transplantation |
| OPS | 5-467.6 | Small intestine transplant | Transplantation |
| OPS | 8-979 | Inpatient treatment before transplantation | Transplantation |
| OPS | 8-97c | Inpatient treatment after admission to the organ transplant waiting list | Transplantation |
| OPS | 5-525 | (Total) pancreatectomy | Other |
| ICD-10-GM | C91 | Lymphatic leukemia | Leukemia |
| ICD-10-GM | C92 | Myeloid Leukemia | Leukemia |
| ICD-10-GM | C93 | Monocytic leukemia | Leukemia |
| ICD-10-GM | C94 | Other leukemias of specified cell type | Leukemia |
| ICD-10-GM | C95 | Leukemia, unspecified | Leukemia |

Abbreviations: HIV, Human Immunodeficiency Virus [Humane Immundefizienz-Viruskrankheit]; HU, High Urgency; ICD-10-GM, International Classification of Diseases, 10th Revision, German Modification; OPS, Operation and Procedure Codes [Operationen- und Prozedurenschlüssel].

Table S2. cCMV-specific Symptoms and Sequelae

| Type | Code | Description | Group |
| --- | --- | --- | --- |
| ICD-10-GM | A04 | Other bacterial intestinal infections | Intestinal disorders |
| ICD-10-GM | A09 | Infectious gastroenteritis and colitis, unspecified | Intestinal disorders |
| ICD-10-GM | D61.0 | Constitutional aplastic anemia | Anemia, neutropenia |
| ICD-10-GM | D61.3 | Idiopathic aplastic anemia | Anemia, neutropenia |
| ICD-10-GM | D61.8 | Other specified aplastic anemias and other bone marrow failure syndromes | Anemia, neutropenia |
| ICD-10-GM | D61.9 | Aplastic anemia, unspecified | Anemia, neutropenia |
| ICD-10-GM | D69 | Purpura and other hemorrhagic conditions | Purpura |
| ICD-10-GM | D70.0 | Congenital agranulocytosis | Anemia, neutropenia |
| ICD-10-GM | D70.5 | Cyclic neutropenia | Anemia, neutropenia |
| ICD-10-GM | D70.6 | Other neutropenia | Anemia, neutropenia |
| ICD-10-GM | D70.7 | Neutropenia, unspecified | Anemia, neutropenia |
| ICD-10-GM | D89 | Other disorders involving the immune mechanism, not elsewhere classified | Purpura |
| ICD-10-GM | F44.5 | Dissociative seizures | Cerebral seizures |
| ICD-10-GM | F80 | Specific developmental disorders of speech and language | Cognitive developmental disorders |
| ICD-10-GM | F81 | Specific developmental disorders of scholastic skills | Cognitive developmental disorders |
| ICD-10-GM | F82 | Specific developmental disorder of motor function | Motor development disorders |
| ICD-10-GM | F83 | Combined circumscribed developmental disorders | Motor development disorders |
| ICD-10-GM | F84 | Pervasive developmental disorders | Motor development disorders |
| ICD-10-GM | F88 | Other disorders of psychological development | Cognitive developmental disorders |
| ICD-10-GM | F89 | Unspecified disorder of psychological development | Cognitive developmental disorders |
| ICD-10-GM | G40 | Epilepsy | Cerebral seizures |
| ICD-10-GM | G41 | Status epilepticus | Cerebral seizures |
| ICD-10-GM | G80 | Infantile cerebral palsy | Paralysis |
| ICD-10-GM | G81 | Hemiplegia and hemiparesis | Paralysis |
| ICD-10-GM | G82 | Paraparesis and paraplegia, tetraparesis and tetraplegia | Paralysis |
| ICD-10-GM | G83 | Other paralytic syndromes | Paralysis |
| ICD-10-GM | H30 | Chorioretinitis | Chorioretinitis including retinal scar |
| ICD-10-GM | H31.0 | Chorioretinal scars | Chorioretinitis including retinal scar |
| ICD-10-GM | H47.2 | Optic atrophy | Optic atrophy |
| ICD-10-GM | H48.0 | Optic atrophy in diseases classified elsewhere | Optic atrophy |
| ICD-10-GM | H53 | Visual disturbances | Loss of vision |
| ICD-10-GM | H54 | Blindness and low vision | Loss of vision |
| ICD-10-GM | H90 | Conductive and sensorineural hearing loss | Sensorineural hearing loss to deafness (newborn hearing screening) |
| ICD-10-GM | H91 | Other hearing loss | Sensorineural hearing loss to deafness (newborn hearing screening) |
| ICD-10-GM | M31.1 | Thrombotic microangiopathy | Purpura |
| ICD-10-GM | P05 | Intrauterine deficiency development and fetal malnutrition | Intrauterine growth retardation |
| ICD-10-GM | P07.0 | Extremely low birth weight newborn | Intrauterine growth retardation |
| ICD-10-GM | P07.1 | Other low birth weight newborn | Intrauterine growth retardation |
| ICD-10-GM | P07.2 | Extreme immaturity of newborn (<28 week of pregnancy) | Prematurity |
| ICD-10-GM | P07.3 | Preterm [premature] newborn [other] (28-36 week of pregnancy) | Prematurity |
| ICD-10-GM | P23 | Congenital pneumonia | Pneumonia |
| ICD-10-GM | P54.5 | Skin bleeding in the newborn (ecchymoses, petechiae) | Disseminated petechiae |
| ICD-10-GM | P59 | Neonatal icterus from other and unspecified causes | Verdinikterus (direct hyperbilirubinemia) |
| ICD-10-GM | P60 | Disseminated intravascular coagulation of newborn | Purpura |
| ICD-10-GM | P61.0 | Transient thrombocytopenia in the newborn | Thrombocytopenia |
| ICD-10-GM | P61.2 | Anemia of prematurity | Anemia, neutropenia |
| ICD-10-GM | P61.4 | Other congenital anemias, not elsewhere classified | Anemia, neutropenia |
| ICD-10-GM | P61.5 | Transient neonatal neutropenia | Anemia, neutropenia |
| ICD-10-GM | P77 | Necrotizing enterocolitis of newborn | Intestinal disorders |
| ICD-10-GM | P90 | Convulsions of newborn | Cerebral seizures |
| ICD-10-GM | P91 | Other disturbances of cerebral status of newborn | Cerebral seizures |
| ICD-10-GM | Q00 | Anencephaly and similar malformations | Migration disorders of the CNS |
| ICD-10-GM | Q01 | Encephalocele | Migration disorders of the CNS |
| ICD-10-GM | Q02 | Microcephaly | Migration disorders of the CNS |
| ICD-10-GM | Q03 | Congenital hydrocephalus | Migration disorders of the CNS |
| ICD-10-GM | Q04 | Other congenital malformations of brain | Migration disorders of the CNS |
| ICD-10-GM | Q05 | Spina bifida | Migration disorders of the CNS |
| ICD-10-GM | Q06 | Other congenital malformations of spinal cord | Migration disorders of the CNS |
| ICD-10-GM | Q07 | Other congenital malformations of nervous system | Migration disorders of the CNS |
| ICD-10-GM | R16 | Hepatomegaly and splenomegaly, not elsewhere classified | Hepatosplenomegaly |
| ICD-10-GM | R23.3 | Spontaneous ecchymoses | Disseminated petechiae |
| ICD-10-GM | R56 | Convulsions, not elsewhere classified | Cerebral seizures |
| ICD-10-GM | R83 | Abnormal findings in cerebrospinal fluid | Abnormal findings in cerebrospinal fluid |

Abbreviations: cCMV, congenital cytomegalovirus; CNS, central nervous system; ICD-10-GM, International Classification of Diseases, 10th Revision, German Modification.

Table S3. All-cause Healthcare Costs^a^ During the First 1-365 Days of Life After Winsorization

| Cost Domain | cCMV_90_ cohort | “Representative” controls | Mean difference (CI) | p-value^b^ | cCMV_21-S_ cohort | “Healthy” controls | Mean difference (CI) | p-value^b^ |
| --- | --- | --- | --- | --- | --- | --- | --- | --- |
| **Outpatient care** |  |  |  |  |  |  |  |  |
| Sum | 52,012 | 2,006,609 |  |  | 24,532 | 748,140 |  |  |
| **Mean** | **963** | **619** | **344 (248-439)** | **<0.01** | **1,022** | **520** | **503 (345-661)** | **<0.01** |
| SD | 358 | 175 |  |  | 395 | 119 |  |  |
| Min | 328 | 0 |  |  | 328 | 214 |  |  |
| Q1 | 695 | 505 |  |  | 818 | 440 |  |  |
| Median | 947 | 604 |  |  | 1,009 | 516 |  |  |
| Q3 | 1,171 | 721 |  |  | 1,244 | 589 |  |  |
| Max | 1,643 | 994 |  |  | 1,640 | 994 |  |  |
| **Inpatient care** |  |  |  |  |  |  |  |  |
| Sum | 909,279 | 3,465,073 |  |  | 592,880 | 141,126 |  |  |
| **Mean** | **16,839** | **1,069** | **15,769 (9,660-21,878)** | **<0.01** | **24,703** | **98** | **24,605 (15,503-33,708)** | **<0.01** |
| SD | 22,901 | 2,141 |  |  | 22,751 | 547 |  |  |
| Min | 0 | 0 |  |  | 3,238 | 0 |  |  |
| Q1 | 3,683 | 0 |  |  | 10,531 | 0 |  |  |
| Median | 8,343 | 0 |  |  | 14,723 | 0 |  |  |
| Q3 | 19,407 | 986 |  |  | 29,972 | 0 |  |  |
| Max | 87,970 | 8,011 |  |  | 82,699 | 8,011 |  |  |
| **Pharmaceuticals** |  |  |  |  |  |  |  |  |
| Sum | 55,524 | 261,083 |  |  | 28,974 | 57,184 |  |  |
| **Mean** | **1,028** | **81** | **948 (518-1,377)** | **<0.01** | **1,207** | **40** | **1,168 (531-1,804)** | **<0.01** |
| SD | 1,611 | 75 |  |  | 1,590 | 40 |  |  |
| Min | 1 | 0 |  |  | 6 | 0 |  |  |
| Q1 | 71 | 29 |  |  | 85 | 15 |  |  |
| Median | 154 | 54 |  |  | 503 | 29 |  |  |
| Q3 | 1,184 | 103 |  |  | 1,434 | 49 |  |  |
| Max | 5,342 | 293 |  |  | 5,082 | 293 |  |  |
| **Aids & Remedies^c^** |  |  |  |  |  |  |  |  |
| Sum | 32,262 | 333,203 |  |  | 22,019 | 17,508 |  |  |
| **Mean** | **597** | **103** | **495 (257-733)** | **<0.01** | 917 | **12** | **905 (428-1,383)** | **<0.01** |
| SD | 892 | 133 |  |  | 1,193 | 50 |  |  |
| Min | 0 | 0 |  |  | 0 | 0 |  |  |
| Q1 | 0 | 0 |  |  | 0 | 0 |  |  |
| Median | 208 | 29 |  |  | 394 | 0 |  |  |
| Q3 | 850 | 165 |  |  | 1,487 | 13 |  |  |
| Max | 3,580 | 508 |  |  | 3,580 | 508 |  |  |
| **Total** |  |  |  |  |  |  |  |  |
| Sum | 1,054,717 | 6,374,032 |  |  | 675,667 | 969,832 |  |  |
| **Mean** | **19,532** | **1,967** | **17,565 (11,100-24,029)** | **<0.01** | **28,153** | **673** | **27,479 (17,992-36,967)** | **<0.01** |
| SD | 24,235 | 2,440 |  |  | 23,713 | 625 |  |  |
| Min | 655 | 0 |  |  | 5,015 | 214 |  |  |
| Q1 | 4,958 | 653 |  |  | 12,950 | 479 |  |  |
| Median | 9,759 | 863 |  |  | 20,924 | 569 |  |  |
| Q3 | 21,623 | 2,106 |  |  | 31,201 | 667 |  |  |
| Max | 94,039 | 9,997 |  |  | 89,011 | 9,997 |  |  |

Abbreviations: cCMV, congenital cytomegalovirus; cCMV_21-S_, infants with inpatient cCMV diagnosis and symptoms during the first 21 days of life; cCMV_90_, infants with cCMV diagnosis during the first 90 days of life; CI, 95% confidence interval; “Healthy”, infants with no ICD-10-GM diagnosis (except Z-diagnoses) until 4^th^ preventive health checkup and no cCMV or CMV diagnosis in the observation period; Max, maximum; Min, minimum; Q1, 25^th^ percentile; Q3, 75^th^ percentile; “Representative”, infants with no cCMV or CMV diagnosis in the observation period; SD, standard deviation.

^a^ Costs are displayed in Euros (€). Figures were commercially rounded, which may result in minor calculation differences.

^b^ P-value <0.05 was considered as statistically significant (Wilcoxon rank-sum test).

^c^ Data for aids and remedies were not completely available for all individuals in the database (18.5% cCMV_90_ cohort and 31.6% respective controls, 12.5% cCMV_21-S_ cohort and 25.9% respective controls), and single imputation was applied using the mean costs of infants with available data.

Table S4. All-cause Healthcare Costs^a^ During the First 366-730 Days of Life After Winsorization

| Cost Domain | cCMV_90_ cohort | “Representative” controls | Mean difference (CI) | p-value^b^ | cCMV_21-S_ cohort | “Healthy” controls | Mean difference (CI) | p-value^b^ |
| --- | --- | --- | --- | --- | --- | --- | --- | --- |
| **Outpatient** |  |  |  |  |  |  |  |  |
| Sum | 21,296 | 883,373 |  |  | 9,167 | 326,167 |  |  |
| **Mean** | **626** | **433** | **193 (104-283)** | **<0.01** | **611** | **362** | **249 (130-368)** | **<0.01** |
| SD | 266 | 172 |  |  | 235 | 149 |  |  |
| Min | 235 | 0 |  |  | 235 | 0 |  |  |
| Q1 | 400 | 313 |  |  | 470 | 265 |  |  |
| Median | 622 | 414 |  |  | 654 | 350 |  |  |
| Q3 | 792 | 533 |  |  | 774 | 444 |  |  |
| Max | 1,113 | 810 |  |  | 972 | 810 |  |  |
| **Inpatient** |  |  |  |  |  |  |  |  |
| Sum | 49,834 | 450,378 |  |  | 12,812 | 126,520 |  |  |
| **Mean** | **1,466** | **221** | **1,245 (402-2,088)** | **<0.01** | **854** | **141** | **714 (146-1,281)** | **<0.01** |
| SD | 2,507 | 585 |  |  | 1,120 | 476 |  |  |
| Min | 0 | 0 |  |  | 0 | 0 |  |  |
| Q1 | 0 | 0 |  |  | 0 | 0 |  |  |
| Median | 452 | 0 |  |  | 485 | 0 |  |  |
| Q3 | 1,841 | 0 |  |  | 1,325 | 0 |  |  |
| Max | 9,108 | 2,152 |  |  | 4,050 | 2,152 |  |  |
| **Pharmaceuticals** |  |  |  |  |  |  |  |  |
| Sum | 3,214 | 151,781 |  |  | 1,012 | 44,387 |  |  |
| **Mean** | **95** | **74** | **20 (-18-59)** | **0.85** | **67** | **49** | **18 (-15-52)** | **0.17** |
| SD | 114 | 70 |  |  | 66 | 56 |  |  |
| Min | 0 | 0 |  |  | 0 | 0 |  |  |
| Q1 | 27 | 24 |  |  | 25 | 11 |  |  |
| Median | 50 | 51 |  |  | 40 | 32 |  |  |
| Q3 | 95 | 101 |  |  | 92 | 66 |  |  |
| Max | 408 | 261 |  |  | 219 | 261 |  |  |
| **Aids & Remedies^c^** |  |  |  |  |  |  |  |  |
| Sum | 40,982 | 107,525 |  |  | 19,687 | 34,023 |  |  |
| **Mean** | **1,205** | **53** | **1,153 (329-1,976)** | **0.42** | **1,312** | **38** | **1,275 (8-2,541)** | **0.20** |
| SD | 2,451 | 63 |  |  | 2,502 | 49 |  |  |
| Min | 0 | 0 |  |  | 0 | 0 |  |  |
| Q1 | 0 | 0 |  |  | 0 | 0 |  |  |
| Median | 0 | 0 |  |  | 0 | 0 |  |  |
| Q3 | 1,374 | 131 |  |  | 1,418 | 97 |  |  |
| Max | 7,970 | 131 |  |  | 7,423 | 131 |  |  |
| **Total** |  |  |  |  |  |  |  |  |
| Sum | 120,693 | 1,698,336 |  |  | 41,740 | 552,861 |  |  |
| **Mean** | **3,550** | **833** | **2,717 (1,076-4,359)** | **<0.01** | **2,783** | **614** | **2,168 (562-3,775)** | **<0.01** |
| SD | 4,883 | 790 |  |  | 3,173 | 613 |  |  |
| Min | 253 | 0 |  |  | 253 | 7 |  |  |
| Q1 | 618 | 404 |  |  | 993 | 323 |  |  |
| Median | 1,464 | 562 |  |  | 1,977 | 441 |  |  |
| Q3 | 3,728 | 809 |  |  | 2,537 | 605 |  |  |
| Max | 17,488 | 3,344 |  |  | 11,413 | 3,344 |  |  |

Abbreviations: cCMV, congenital cytomegalovirus; cCMV_21-S_, infants with inpatient cCMV diagnosis and symptoms during the first 21 days of life; cCMV_90_, infants with cCMV diagnosis during the first 90 days of life; CI, 95% confidence interval; “Healthy”, infants with no ICD-10-GM diagnosis (except Z-diagnoses) until 4^th^ preventive health checkup and no cCMV or CMV diagnosis in the observation period; Max, maximum; Min, minimum; Q1, 25^th^ percentile; Q3, 75^th^ percentile; “Representative”, infants with no cCMV or CMV diagnosis in the observation period; SD, standard deviation.

^a^ Costs are displayed in Euros (€). Figures were commercially rounded, which may result in minor calculation differences.

^b^ P-value <0.05 was considered as statistically significant (Wilcoxon rank-sum test).

^c^ Data for aids and remedies were not completely available for all individuals in the database (20.6% cCMV_90_ cohort and 34.0% respective controls, 20.0% cCMV_21-S_ cohort and 33.0% respective controls), and single imputation was applied using the mean costs of infants with available data.

1. In Germany, ten preventive health checkups for newborns are available free of charge for all newborns and infants and are scheduled for different time periods. The 1^st^ checkup takes place immediately after birth, 2^nd^ checkup between 3.-10. day of life, 3^rd^ checkup between 4.-5. week of life, 4^th^ checkup between 3.-4. month of life. (<https://www.kindergesundheit-info.de/themen/ernaehrung/frueherkennung-u1-u9-und-j1/untersuchungen-u1-bis-u9/die-untersuchungen-u1-bis-u9/>) [↑](#footnote-ref-2)
