# Supplementary material for: Healthcare costs of congenital cytomegalovirus (cCMV) disease in infants during the first two years of life: a retrospective German claims database analysis
Source: Cost Eff Resour Alloc. 2023 Jan 23;21:8. doi: 10.1186/s12962-022-00411-x (PMC9872342; doi:10.1186/s12962-022-00411-x)
Supplement: Supplementary file 2 — Additional file 2. Table S1. Baseline Demographic and Clinical Characteristicsa After Matching–Complementary Comparisons. Table S2. All-cause Healthcare Costsa During the First 1-365 Days of Life, Unadjusted–Complementary Comparisons. Table S3. All-cause Healthcare Costsa During the First 366-730 Days of Life, Unadjusted – Complementary Comparisons. Table S4. All-cause Healthcare Costsa During the First 1-365 Days of Life After Winsorization – Complementary Comparisons. Table S5. All-cause Healthcare Costsa During the First 366-730 Days of Life After Winsorization–Complementary Comparisons. [file 12962_2022_411_MOESM2_ESM.docx]

# Additional file 2

In the following, we present the results of the complementary comparisons of cCMV cohorts and control groups, which have not been presented in the manuscript. The complementary comparisons include the comparison of cCMV_90_ cohort vs. “healthy” controls and cCMV_21-S_ cohort vs. “representative” controls.

Table S1. Baseline Demographic and Clinical Characteristics^a^ After Matching – Complementary Comparisons

| Characteristic | cCMV_90_ cohort  N (%) | “Healthy” controls  N (%) | cCMV_21-S_ cohort  N (%) | “Representative” controls N (%) |
| --- | --- | --- | --- | --- |
| Observability |  |  |  |  |
| 1-365 days of life | 54 (100.0) | 3,240 (100.0) | 24 (100.0) | 1,440 (100.0) |
| 366-730 days of life | 34 (63.0) | 2,040 (63.0) | 15 (62.5) | 900 (62.5) |
| Gender |  |  |  |  |
| Male | 30 (55.6) | 1,800 (55.6) | 15 (62.5) | 900 (62.5) |
| Female | 24 (44.4) | 1,440 (44.4) | 9 (37.5) | 540 (37.5) |
| Birth quarter |  |  |  |  |
| Q1 | 11 (20.4) | 660 (20.4) | 6 (25.0) | 360 (25.0) |
| Q2 | 14 (25.9) | 840 (25.9) | 7 (29.2) | 420 (29.2) |
| Q3 | 14 (25.9) | 840 (25.9) | <5 (-) | 180 (12.5) |
| Q4 | 15 (27.8) | 900 (27.8) | 8 (33.3) | 480 (33.3) |
| Birth weight |  |  |  |  |
| Extremely low (P07.0-) | <5 (-) | 0 (0.0) | <5 (-) | <5 (-) |
| Low (P07.1-) | 14 (25.9) | 0 (0.0) | 12 (50.0) | 74 (5.1) |
| High (P08.1 or P08.2) | 0 (0.0) | 0 (0.0) | 0 (0.0) | 8 (0.6) |
| Normal (infants with none of the stated codes) | 37 (68.5) | 3,240 (100.0) | 9 (37.5) | 1,357 (94.2) |
| Region |  |  |  |  |
| North^b^ | 10 (18.5) | 563 (17.4) | <5 (-) | 221 (15.3) |
| East^c^ | 9 (16.7) | 259 (8.0) | 7 (29.2) | 149 (10.3) |
| West^d^ | 21 (38.9) | 1,153 (35.6) | 8 (33.3) | 534 (37.1) |
| South^e^ | 14 (25.9) | 1,262 (39.0) | 7 (29.2) | 533 (37.0) |
| Unknown | 0 (0.0) | <5 (-) | 0 (0.0) | <5 (-) |

Abbreviations: cCMV, congenital cytomegalovirus; cCMV_21-S_, infants with inpatient cCMV diagnosis and symptoms during the first 21 days of life; cCMV_90_, infants with cCMV diagnosis during the first 90 days of life; “Healthy”, infants with no ICD-10-GM diagnosis (except Z-diagnoses) until 4^th^ preventive health checkup and no cCMV or CMV diagnosis in the observation period; Q1, January 1^st^–March 31^st^; Q2, April 1^st^–June 30^th^; Q3, July 1^st^–September 30^th^; Q4, October 1^st^–December 31^st^; “Representative”, infants with no cCMV or CMV diagnosis in the observation period.

^a^ Baseline demographic and clinical characteristics were assessed during the first 1-365 days of life.

^b^ North includes federal states Schleswig-Holstein, Hamburg, Bremen, Lower Saxony, and Mecklenburg-Western Pomerania.

^c^ East includes federal states Thuringia, Brandenburg, Berlin, Saxony, and Saxony-Anhalt.

^d^ West includes federal states North Rhine-Westphalia, Saarland, Rhineland-Palatinate, and Hesse.

^e^ South includes federal states Bavaria and Baden-Wuerttemberg.

Table S2. All-cause Healthcare Costs^a^ During the First 1-365 Days of Life, Unadjusted – Complementary Comparisons

| Cost Domain | cCMV_90_ cohort | “Healthy” controls | Mean difference (CI) | p-value**^b^** | cCMV_21-S_ cohort | “Representative” controls | Mean difference (CI) | p-value**^b^** |
| --- | --- | --- | --- | --- | --- | --- | --- | --- |
| **Outpatient** |  |  |  |  |  |  |  |  |
| Sum | 52,563 | 1,693,974 |  |  | 25,007 | 909,942 |  |  |
| **Mean** | **973** | **523** | **451**  **(349-552)** | **<0.01** | **1,042** | **632** | **410**  **(236-585)** | **<0.01** |
| SD | 382 | 123 |  |  | 435 | 223 |  |  |
| Min | 328 | 108 |  |  | 328 | 0 |  |  |
| Q1 | 695 | 441 |  |  | 818 | 503 |  |  |
| Median | 947 | 515 |  |  | 1,009 | 600 |  |  |
| Q3 | 1,171 | 590 |  |  | 1,244 | 732 |  |  |
| Max | 2,082 | 1,843 |  |  | 2,082 | 2,986 |  |  |
| **Inpatient** |  |  |  |  |  |  |  |  |
| Sum | 1,071,210 | 374,098 |  |  | 737,247 | 3,039,448 |  |  |
| **Mean** | **19,837** | **115** | **19,722**  **(10,505-28,939)** | **<0.01** | **30,719** | **2,111** | **28,608**  **(11,630-45,586)** | **<0.01** |
| SD | 34,555 | 954 |  |  | 42,425 | 7,839 |  |  |
| Min | 0 | 0 |  |  | 3,238 | 0 |  |  |
| Q1 | 3,683 | 0 |  |  | 10,531 | 0 |  |  |
| Median | 8,343 | 0 |  |  | 14,723 | 0 |  |  |
| Q3 | 19,407 | 0 |  |  | 29,972 | 1,114 |  |  |
| Max | 194,846 | 35,922 |  |  | 194,846 | 171,846 |  |  |
| **Pharmaceuticals** |  |  |  |  |  |  |  |  |
| Sum | 58,093 | 137,759 |  |  | 30,013 | 194,051 |  |  |
| **Mean** | **1,076** | **43** | **1,033**  **(565-1,502)** | **<0.01** | **1,251** | **135** | **1,116**  **(433-1,799)** | **<0.01** |
| SD | 1,757 | 85 |  |  | 1,705 | 676 |  |  |
| Min | 1 | 0 |  |  | 6 | 0 |  |  |
| Q1 | 71 | 15 |  |  | 85 | 28 |  |  |
| Median | 154 | 28 |  |  | 503 | 50 |  |  |
| Q3 | 1,184 | 49 |  |  | 1,434 | 99 |  |  |
| Max | 7,272 | 2,779 |  |  | 5,903 | 16,515 |  |  |
| **Aids & Remedies^c^** |  |  |  |  |  |  |  |  |
| Sum | 45,924 | 56,276 |  |  | 35,681 | 220,396 |  |  |
| **Mean** | **850** | **17** | **833**  **(324-1,343)** | **<0.01** | **1,487** | **153** | **1,334**  **(243-2,425)** | **<0.01** |
| SD | 1,910 | 85 |  |  | 2,726 | 637 |  |  |
| Min | 0 | 0 |  |  | 0 | 0 |  |  |
| Q1 | 0 | 0 |  |  | 0 | 0 |  |  |
| Median | 208 | 0 |  |  | 394 | 0 |  |  |
| Q3 | 850 | 17 |  |  | 1,487 | 153 |  |  |
| Max | 10,694 | 2,910 |  |  | 10,694 | 13,322 |  |  |
| **Total** |  |  |  |  |  |  |  |  |
| Sum | 1,227,790 | 2,262,107 |  |  | 827,948 | 4,363,836 |  |  |
| **Mean** | **22,737** | **698** | **22,039**  **(12,213-31,864)** | **<0.01** | **34,498** | **3,030** | **31,467**  **(13,455-49,480)** | **<0.01** |
| SD | 36,838 | 988 |  |  | 45,008 | 8,551 |  |  |
| Min | 655 | 162 |  |  | 5,015 | 0 |  |  |
| Q1 | 4,958 | 485 |  |  | 12,950 | 635 |  |  |
| Median | 9,759 | 570 |  |  | 20,924 | 843 |  |  |
| Q3 | 21,623 | 668 |  |  | 31,201 | 2,220 |  |  |
| Max | 212,552 | 36,901 |  |  | 212,552 | 186,005 |  |  |

Abbreviations: cCMV, congenital cytomegalovirus; cCMV_21-S_, infants with inpatient cCMV diagnosis and symptoms during the first 21 days of life; cCMV_90_, infants with cCMV diagnosis during the first 90 days of life; “Healthy”, infants with no ICD-10-GM diagnosis (except Z-diagnoses) until 4^th^ preventive health checkup and no cCMV or CMV diagnosis in the observation period; Max, maximum; Min, minimum; Q1, 25^th^ percentile; Q3, 75^th^ percentile; “Representative”, infants with no cCMV or CMV diagnosis in the observation period; CI, 95% confidence interval; SD, standard deviation.

^a^ Costs are displayed in Euros (€). Figures were commercially rounded, which may result in minor calculation differences.

^b^ P-value <0.05 was considered as statistically significant (Wilcoxon rank-sum test).

^c^ Data for aids and remedies were not completely available for all individuals in the database (18.5% cCMV_90_ cohort and 31.3% respective controls, 12.5% cCMV_21-S_ cohort and 25.8% respective controls), and single imputation was applied using the mean costs of infants with available data.

Table S3. All-cause Healthcare Costs^a^ During the First 366-730 Days of Life, Unadjusted – Complementary Comparisons

| Cost Domain | cCMV_90_ cohort | “Healthy” controls | Mean difference (CI) | p-value**^b^** | cCMV_21-S_ cohort | “Representative” controls | Mean difference (CI) | p-value**^b^** |
| --- | --- | --- | --- | --- | --- | --- | --- | --- |
| **Outpatient** |  |  |  |  |  |  |  |  |
| Sum | 22,386 | 759,856 |  |  | 9,295 | 396,116 |  |  |
| **Mean** | **658** | **372** | **286**  **(162-410)** | **<0.01** | **620** | **440** | **180 (52-307)** | **<0.01** |
| SD | 369 | 170 |  |  | 251 | 221 |  |  |
| Min | 235 | 0 |  |  | 235 | 0 |  |  |
| Q1 | 400 | 269 |  |  | 470 | 305 |  |  |
| Median | 622 | 354 |  |  | 654 | 409 |  |  |
| Q3 | 792 | 446 |  |  | 774 | 526 |  |  |
| Max | 2,179 | 2,186 |  |  | 1,100 | 2,272 |  |  |
| **Inpatient** |  |  |  |  |  |  |  |  |
| Sum | 74,440 | 378,041 |  |  | 39,097 | 300,367 |  |  |
| **Mean** | **2,189** | **185** | **2,004**  **(128-3,880)** | **<0.01** | **2,606** | **334** | **2,273**  **(-1,625-6,171)** | **<0.01** |
| SD | 5,580 | 1,084 |  |  | 7,702 | 1,078 |  |  |
| Min | 0 | 0 |  |  | 0 | 0 |  |  |
| Q1 | 0 | 0 |  |  | 0 | 0 |  |  |
| Median | 452 | 0 |  |  | 485 | 0 |  |  |
| Q3 | 1,841 | 0 |  |  | 1,325 | 0 |  |  |
| Max | 30,335 | 35,703 |  |  | 30,335 | 12,963 |  |  |
| **Pharmaceuticals** |  |  |  |  |  |  |  |  |
| Sum | 3,549 | 117,930 |  |  | 1,052 | 86,509 |  |  |
| **Mean** | **104** | **58** | **47 (-3-96)** | **0.03** | **70** | **96** | **-26**  **(-72-21)** | **0.81** |
| SD | 146 | 146 |  |  | 73 | 437 |  |  |
| Min | 0 | 0 |  |  | 0 | 0 |  |  |
| Q1 | 27 | 14 |  |  | 25 | 25 |  |  |
| Median | 50 | 34 |  |  | 40 | 50 |  |  |
| Q3 | 95 | 68 |  |  | 92 | 93 |  |  |
| Max | 654 | 5,604 |  |  | 260 | 12,509 |  |  |
| **Aids & Remedies^c^** |  |  |  |  |  |  |  |  |
| Sum | 46,714 | 148,600 |  |  | 21,266 | 73,792 |  |  |
| **Mean** | **1,374** | **73** | **1,301**  **(289-2,313)** | **0.30** | **1,418** | **82** | **1,336**  **(-78-2,750)** | **0.29** |
| SD | 3,005 | 1,426 |  |  | 2,794 | 451 |  |  |
| Min | 0 | 0 |  |  | 0 | 0 |  |  |
| Q1 | 0 | 0 |  |  | 0 | 0 |  |  |
| Median | 0 | 0 |  |  | 0 | 0 |  |  |
| Q3 | 1,374 | 73 |  |  | 1,418 | 82 |  |  |
| Max | 12,669 | 64,003 |  |  | 9,002 | 7,253 |  |  |
| **Total** |  |  |  |  |  |  |  |  |
| Sum | 147,089 | 1,404,427 |  |  | 70,711 | 856,783 |  |  |
| **Mean** | **4,326** | **688** | **3,638**  **(994-6,282)** | **<0.01** | **4,714** | **952** | **3,762**  **(-1,343-8,867)** | **<0.01** |
| SD | 7,860 | 2,371 |  |  | 10,087 | 1,413 |  |  |
| Min | 253 | 0 |  |  | 253 | 0 |  |  |
| Q1 | 618 | 322 |  |  | 993 | 401 |  |  |
| Median | 1,464 | 435 |  |  | 1,977 | 530 |  |  |
| Q3 | 3,728 | 587 |  |  | 2,537 | 828 |  |  |
| Max | 40,384 | 100,087 |  |  | 40,384 | 14,794 |  |  |

Abbreviations: cCMV, congenital cytomegalovirus disease; cCMV_21-S_, infants with inpatient cCMV diagnosis and symptoms during the first 21 days of life; cCMV_90_, infants with cCMV diagnosis during the first 90 days of life; CI, 95% confidence interval; “Healthy”, infants with no ICD-10-GM diagnosis (except Z-diagnoses) until 4^th^ preventive health checkup and no cCMV or CMV diagnosis in the observation period; Max, maximum; Min, minimum; Q1, 25^th^ percentile; Q3, 75^th^ percentile; “Representative”, infants with no cCMV or CMV diagnosis in the observation period; SD, standard deviation.

^a^ Costs are displayed in Euros (€). Figures were commercially rounded, which may result in minor calculation differences.

^b^ P-value <0.05 was considered as statistically significant (Wilcoxon rank-sum test).

^c^ Data for aids and remedies were not completely available for all individuals in the database (20.6% cCMV_90_ cohort and 34.0% respective controls, 20.0% cCMV_21-S_ cohort and 32.4% respective controls), and single imputation was applied using the mean costs of infants with available data.

Table S4. All-cause Healthcare Costs^a^ During the First 1-365 Days of Life After Winsorization – Complementary Comparisons

| Cost Domain | cCMV_90_ cohort | “Healthy” controls | Mean difference (CI) | p-value**^b^** | cCMV_21-S_ cohort | “Representative” controls | Mean difference (CI) | p-value**^b^** |
| --- | --- | --- | --- | --- | --- | --- | --- | --- |
| **Outpatient care** |  |  |  |  |  |  |  |  |
| Sum | 52,012 | 1,678,715 |  |  | 24,532 | 890,871 |  |  |
| **Mean** | **963** | **518** | **445 (350-541)** | **<0.01** | **1,022** | **619** | **404 (245-562)** | **<0.01** |
| SD | 358 | 109 |  |  | 395 | 179 |  |  |
| Min | 328 | 108 |  |  | 328 | 0 |  |  |
| Q1 | 695 | 441 |  |  | 818 | 503 |  |  |
| Median | 947 | 515 |  |  | 1,009 | 600 |  |  |
| Q3 | 1,171 | 590 |  |  | 1,244 | 732 |  |  |
| Max | 1,643 | 729 |  |  | 1,640 | 994 |  |  |
| **Inpatient** **care** |  |  |  |  |  |  |  |  |
| Sum | 909,279 | 93,781 |  |  | 592,880 | 1,663,289 |  |  |
| **Mean** | **16,839** | **29** | **16,810 (10,701-22,918)** | **<0.01** | **24,703** | **1,155** | **23,548 (14,445-32,651)** | **<0.01** |
| SD | 22,901 | 112 |  |  | 22,751 | 2,279 |  |  |
| Min | 0 | 0 |  |  | 3,238 | 0 |  |  |
| Q1 | 3,683 | 0 |  |  | 10,531 | 0 |  |  |
| Median | 8,343 | 0 |  |  | 14,723 | 0 |  |  |
| Q3 | 19,407 | 0 |  |  | 29,972 | 1,114 |  |  |
| Max | 87,970 | 499 |  |  | 82,699 | 8,011 |  |  |
| **Pharmaceuticals** |  |  |  |  |  |  |  |  |
| Sum | 55,524 | 119,755 |  |  | 28,974 | 111,396 |  |  |
| **Mean** | **1,028** | **37** | **991 (562-1,421)** | **<0.01** | **1,207** | **77** | **1,130 (494-1,766)** | **<0.01** |
| SD | 1,611 | 30 |  |  | 1,590 | 73 |  |  |
| Min | 1 | 0 |  |  | 6 | 0 |  |  |
| Q1 | 71 | 15 |  |  | 85 | 28 |  |  |
| Median | 154 | 28 |  |  | 503 | 50 |  |  |
| Q3 | 1,184 | 49 |  |  | 1,434 | 99 |  |  |
| Max | 5,342 | 119 |  |  | 5,082 | 293 |  |  |
| **Aids & Remedies^c^** |  |  |  |  |  |  |  |  |
| Sum | 32,262 | 24,169 |  |  | 22,019 | 133,209 |  |  |
| **Mean** | **597** | **7** | **590 (352-828)** | **<0.01** | **917** | **93** | **825 (348-1,302)** | **<0.01** |
| SD | 892 | 10 |  |  | 1,193 | 134 |  |  |
| Min | 0 | 0 |  |  | 0 | 0 |  |  |
| Q1 | 0 | 0 |  |  | 0 | 0 |  |  |
| Median | 208 | 0 |  |  | 394 | 0 |  |  |
| Q3 | 850 | 17 |  |  | 1,487 | 153 |  |  |
| Max | 3,580 | 33 |  |  | 3,580 | 508 |  |  |
| **Total** |  |  |  |  |  |  |  |  |
| Sum | 1,054,717 | 2,005,143 |  |  | 675,667 | 2,927,377 |  |  |
| **Mean** | **19,532** | **619** | **18,913 (12,449-25,377)** | **<0.01** | **28,153** | **2,033** | **26,120 (16,632-35,608)** | **<0.01** |
| SD | 24,235 | 229 |  |  | 23,713 | 2,554 |  |  |
| Min | 655 | 162 |  |  | 5,015 | 0 |  |  |
| Q1 | 4,958 | 485 |  |  | 12,950 | 635 |  |  |
| Median | 9,759 | 570 |  |  | 20,924 | 843 |  |  |
| Q3 | 21,623 | 668 |  |  | 31,201 | 2,220 |  |  |
| Max | 94,039 | 1,358 |  |  | 89,011 | 9,997 |  |  |

Abbreviations: cCMV, congenital cytomegalovirus disease; cCMV_21-S_, infants with inpatient cCMV diagnosis and symptoms during the first 21 days of life; cCMV_90_, infants with cCMV diagnosis during the first 90 days of life; CI, 95% confidence interval; “Healthy”, infants with no ICD-10-GM diagnosis (except Z-diagnoses) until 4^th^ preventive health checkup and no cCMV or CMV diagnosis in the observation period; Max, maximum; Min, minimum; Q1, 25^th^ percentile; Q3, 75^th^ percentile; “Representative”, infants with no cCMV or CMV diagnosis in the observation period; SD, standard deviation.

^a^ Costs are displayed in Euros (€). Figures were commercially rounded, which may result in minor calculation differences.

^b^ P-value <0.05 was considered as statistically significant (Wilcoxon rank-sum test).

^c^ Data for aids and remedies were not completely available for all individuals in the database (18.5% cCMV_90_ cohort and 31.3% respective controls, 12.5% cCMV_21-S_ cohort and 25.8% respective controls), and single imputation was applied using the mean costs of infants with available data.

Table S5. All-cause Healthcare Costs^a^ During the First 366-730 Days of Life After Winsorization – Complementary Comparisons

| Cost Domain | cCMV_90_ cohort | “Healthy” controls | Mean difference (CI) | p-value^b^ | cCMV_21-S_ cohort | “Representative” controls | Mean difference (CI) | p-value^b^ |
| --- | --- | --- | --- | --- | --- | --- | --- | --- |
| **Outpatient** |  |  |  |  |  |  |  |  |
| Sum | 21,296 | 742,982 |  |  | 9,167 | 384,154 |  |  |
| **Mean** | **626** | **364** | **262 (172-352)** | **<0.01** | **611** | **427** | **184 (65-304)** | **<0.01** |
| SD | 266 | 142 |  |  | 235 | 171 |  |  |
| Min | 235 | 0 |  |  | 235 | 0 |  |  |
| Q1 | 400 | 269 |  |  | 470 | 305 |  |  |
| Median | 622 | 354 |  |  | 654 | 409 |  |  |
| Q3 | 792 | 446 |  |  | 774 | 526 |  |  |
| Max | 1,113 | 657 |  |  | 972 | 810 |  |  |
| **Inpatient** |  |  |  |  |  |  |  |  |
| Sum | 49,834 | 196,097 |  |  | 12,812 | 212,583 |  |  |
| **Mean** | **1,466** | **96** | **1,370 (527-2,212)** | **<0.01** | **854** | **236** | **618 (50-1,186)** | **<0.01** |
| SD | 2,507 | 332 |  |  | 1,120 | 599 |  |  |
| Min | 0 | 0 |  |  | 0 | 0 |  |  |
| Q1 | 0 | 0 |  |  | 0 | 0 |  |  |
| Median | 452 | 0 |  |  | 485 | 0 |  |  |
| Q3 | 1,841 | 0 |  |  | 1,325 | 0 |  |  |
| Max | 9,108 | 1,386 |  |  | 4,050 | 2,152 |  |  |
| **Pharmaceuticals** |  |  |  |  |  |  |  |  |
| Sum | 3,214 | 99,469 |  |  | 1,012 | 64,269 |  |  |
| **Mean** | **95** | **49** | **46 (7-84)** | **0.02** | **67** | **71** | **-4 (-37-30)** | **0.79** |
| SD | 114 | 48 |  |  | 66 | 67 |  |  |
| Min | 0 | 0 |  |  | 0 | 0 |  |  |
| Q1 | 27 | 14 |  |  | 25 | 25 |  |  |
| Median | 50 | 34 |  |  | 40 | 50 |  |  |
| Q3 | 95 | 68 |  |  | 92 | 93 |  |  |
| Max | 408 | 177 |  |  | 219 | 261 |  |  |
| **Aids & Remedies^c^** |  |  |  |  |  |  |  |  |
| Sum | 40,982 | 57,822 |  |  | 19,687 | 31,820 |  |  |
| **Mean** | **1,205** | **28** | **1,177 (353-2,001)** | **0.29** | **1,312** | **35** | **1,277 (11-2,543)** | **0.27** |
| SD | 2,451 | 35 |  |  | 2,502 | 44 |  |  |
| Min | 0 | 0 |  |  | 0 | 0 |  |  |
| Q1 | 0 | 0 |  |  | 0 | 0 |  |  |
| Median | 0 | 0 |  |  | 0 | 0 |  |  |
| Q3 | 1,374 | 73 |  |  | 1,418 | 82 |  |  |
| Max | 7,970 | 73 |  |  | 7,423 | 131 |  |  |
| **Total** |  |  |  |  |  |  |  |  |
| Sum | 120,693 | 1,141,534 |  |  | 41,740 | 746,873 |  |  |
| **Mean** | **3,550** | **560** | **2,990 (1,349-4,632)** | **<0.01** | **2,783** | **830** | **1,953 (346-3,559)** | **<0.01** |
| SD | 4,883 | 446 |  |  | 3,173 | 808 |  |  |
| Min | 253 | 0 |  |  | 253 | 0 |  |  |
| Q1 | 618 | 322 |  |  | 993 | 401 |  |  |
| Median | 1,464 | 435 |  |  | 1,977 | 530 |  |  |
| Q3 | 3,728 | 587 |  |  | 2,537 | 828 |  |  |
| Max | 17,488 | 2,094 |  |  | 11,413 | 3,344 |  |  |

Abbreviations: cCMV, congenital cytomegalovirus disease; cCMV_21-S_, infants with inpatient cCMV diagnosis and symptoms during the first 21 days of life; cCMV_90_, infants with cCMV diagnosis during the first 90 days of life; CI, 95% confidence interval; “Healthy”, infants with no ICD-10-GM diagnosis (except Z-diagnoses) until 4^th^ preventive health checkup and no cCMV or CMV diagnosis in the observation period; Max, maximum; Min, minimum; Q1, 25^th^ percentile; Q3, 75^th^ percentile; “Representative”, infants with no cCMV or CMV diagnosis in the observation period; SD, standard deviation.

^a^ Costs are displayed in Euros (€). Figures were commercially rounded, which may result in minor calculation differences.

^b^ P-value <0.05 was considered as statistically significant (Wilcoxon rank-sum test).

^c^ Data for aids and remedies were not completely available for all individuals in the database (20.6% cCMV_90_ cohort and 34.0% respective controls, 20.0% cCMV_21-S_ cohort and 32.4% respective controls), and single imputation was applied using the mean costs of infants with available data.
